# Direct confirmation of quiescence of CD34+CD38- leukemia stem cell populations using single cell culture, their molecular signature and clinicopathological implications

**DOI:** 10.1186/s12885-015-1233-x

**Published:** 2015-04-02

**Authors:** Eun Jeong Won, Hye-Ran Kim, Ra-Young Park, Seok-Yong Choi, Jong Hee Shin, Soon-Pal Suh, Dong-Wook Ryang, Michael Szardenings, Myung-Geun Shin

**Affiliations:** 1 Department of Laboratory Medicine, Chonnam National University Medical School and Chonnam National University Hwasun Hospital, Hwasun, South Korea; 2 Brain Korea 21 Project, Center for Biomedical Human Resources, Chonnam National University, Gwangju, South Korea; 3 Environment Health Center for Childhood Leukemia and Cancer, Chonnam National University Hwasun Hospital, Hwasun, South Korea; 4 Department of Cell Therapy, Fraunhofer Institute for Cell Therapy and Immunology, Leipzig, Germany; 5 College of Korean Medicine, Dongshin University, Naju, South Korea

**Keywords:** CD34+CD38- AML cell, Quiescence, Molecular signature, Prognostic value

## Abstract

**Background:**

The proliferating activity of a single leukemia stem cell and the molecular mechanisms for their quiescent property remain unknown, and also their prognostic value remains a matter of debate. Therefore, this study aimed to demonstrate the quiescence property and molecular signature of leukemia stem cell and their clinicopathological implications.

**Methods:**

Single cell sorting and culture were performed in the various sets of hematopoietic stem cells including CD34+CD38- acute myeloid leukemia (AML) cell population (ASCs) from a total of 60 patients with AML, and 11 healthy controls. Their quiescence related-molecular signatures and clinicopathological parameters were evaluated in AML patients.

**Results:**

Single cell plating efficiency of ASCs was significantly lower (8.6%) than those of normal hematopoietic stem cells i.e.: cord blood, 79.0%; peripheral blood, 45.3%; and bone marrow stem cell, 31.1%. Members of the TGFβ super-family signaling pathway were most significantly decreased; as well as members of the Wnt, Notch, pluripotency maintenance and hedgehog pathways, compared with non ASC populations. mtDNA copy number of ASCs was significantly lower than that of corresponding other cell populations. However, our data couldn’t support the prognostic value of the ASCs in AML.

**Conclusions:**

ASCs showed remarkable lower plating efficiency and slower dividing properties at the single cell level. This quiescence is represented as a marked decrease in the mtDNA copy number and also linked with down-regulation of genes in various molecular pathways.

**Electronic supplementary material:**

The online version of this article (doi:10.1186/s12885-015-1233-x) contains supplementary material, which is available to authorized users.

## Background

Acute myeloid leukemia (AML) is the most common adult leukemia, characterized as a genetically and phenotypically heterogeneous disease [1]. Although AML is generally regarded as a stem-cell disease, there is an ongoing debate on whether normal stem cells undergoing leukemogenic mutations are the cause of leukemogenesis [2]. Since Lapidot *et al.* proposed the concept of leukemia stem cells [3], many researchers demonstrated that leukemic stem-like cells have crucial role in oncogenesis, treatment and prognosis of AML [4-6]. In CD34+ AML, the CD34+ leukemic stem cells designated into AML stem cells (ASCs) are characterized by the absence of CD38 [3,4]. In spite of only a minority of cells within AML, these ASCs are responsible for sustaining and maintaining the leukemia [7]. It has been proven in vitro that these stem cells are more resistant to chemotherapy, compared to the progenitor CD34+CD38+ cells. In vivo, after chemotherapy, the residual malignant CD34+CD38- cells are thought to differentiate, to a limited extent, producing leukemic cells with an immunophenotype, usually observed at diagnosis. Sensitive techniques allow early detection of small numbers of these differentiated leukemic cells, called minimal residual disease; these cells eventually causes relapse of the disease [4]. Therefore, it is important to understand how the biology of the leukemic stem cell in AML differs from normal hematopoietic stem cells.

Hematopoietic stem cells (HSCs) and leukemia stem cells share many features and the extent to which they differ will be the basis for the development of leukemia stem cell-targeted therapies without considerable toxicity. The quiescence of stem cells was regarded to be of critical biologic importance in protecting the stem cell compartment [8]. Quiescence of stem cells might also be a mechanism underlying resistance to cell cycle-dependent cytotoxic therapy [9]. Some researchers examined the gene expression profiles of CD34+CD38- cell populations, compared with CD34+CD38+ cell populations using microarrays and found several different expressions of genes, consistent with the relative quiescence of stem cells [10]. However, the quiescence of ASCs has scarcely been demonstrated at the level of single cell in culture.

Mitochondria, the highly conserved organelles responsible for cellular bioenergetic activity, might play a crucial role in carcinogenesis [11]. Compared to the nuclear genome, mitochondrial DNA (mtDNA) has a modified genetic code, a paucity of introns, and the absence of histone protection. The repair capacity of mtDNA is limited, and the proximity of mtDNA to sites of reactive oxygen species generation suggests that mitochondrial DNA may be more susceptible to mutation than nuclear DNA. Previous studies have shown that mtDNA mutations might be implicated in pathogenesis and/or their prognosis in various malignancies [12-14]. Although stem cells possess lower intracellular mitochondrial contents than other functional mature cells because they generally reside in the G0 phase of the cell cycle and require very little energy [15,16], it is not clear about the mtDNA mutations in terms of the quiescence of ASCs.

AML is maintained by a subpopulation of cancer initiating cells that can regenerate themselves as well as give rise to more differentiated and less proliferative cells that constitute the bulk of the disease. However, there was no comprehensive data regarding the direct confirmation of quiescent characteristics of ASCs on the basis of single cell experiments in vivo and in vitro. The aims of our study were: (i) to demonstrate the quiescence of ASCs at the single cell level, (ii) to elucidate the molecular signature of quiescent ASCs at the nuclear and mitochondrial levels, and (iii) to assign prognostic implications to ASCs in patients with AML.

## Methods

### Study designs and specimens

A total of 60 patients with AML and 11 healthy controls were enrolled after obtaining Chonnam National University Hwasun Hospital’s Institutional Review Board approval and informed consent. The patients who suffered from AML M0 (n = 3), AML M1 (n = 5), AML M2 (n = 34), AML M4 (n = 13), AML M5 (n = 3), and AML M6 (n = 2) were 15 to 82 years aged with a median of 55.5 years. Single cell sorting and culture were performed for the evaluation of plating efficiency in the various sets of hematopoietic stem cells. Plating efficiency of ASCs in bone marrow (BM) obtained from 7 AML patients were compared with that of single normal hematopoietic stem cells, including BM (n = 6), peripheral blood (PB, n = 6) and cord blood (CB, n = 5) which were obtained from healthy controls (n = 11). The samples from the patients and healthy controls were immediately frozen in liquid nitrogen on acquisition, for further molecular evaluation. Their quiescence related-molecular signatures were evaluated in terms of nuclear genomic changes and mtDNA copy number. The clinicopathological parameters in AML patients were also evaluated for prognostic implications of ASCs.

### Single cell sorting for CD34+CD38- cells and CD34+CD38+ cells

The proportion and frequency of ASC were examined using a single cell sorter (BD FACS Aria, BD Biosciences, USA). The samples were lysed by lysing buffer (BD Pharm Lyse, Franklin Lakes, NJ, USA) and incubated at room temperature for 15 minutes; they were then centrifuged for 10 minutes at 1,200 rpm. Then, the cell pellets were washed twice in phosphate-buffered saline (PBS). The number of cells suspended in PBS was adjusted to 2 × 10^6^ cells/mL. Next, 10 μL of anti-CD34 phycoerythrin (PE)–conjugated antibodies (BD Bioscience, Franklin Lakes, NJ, USA) and anti-CD38 fluorescein isothiocyanate (FITC)-conjugated antibodies were added to each 12 × 75 mm tube containing 100 μL of the cell suspension. After incubation for 20 minutes at 4°C, the cells were washed using cold PBS and resuspended in 0.5 mL of buffer. The cell sorting was performed with a FACS aria (BD bioscience, CO, USA) using 100 mW of the 488 nm line of an argon laser (I-90, Coherent, Palo Alto, CA, USA) for excitation. Forward scatter was the triggering parameter. Fluorescence of PE and FITC were detected using a 580/30 band pass filter with gating based on forward scatter and PE and FITC fluorescence, bulk cells of CD34+CD38-(ASC) and CD34+CD38+ cells were collected in a 12 × 75 mm tube containing 100 μL of PBS (Additional file 1).

### Single cell culture and plating efficiency analysis of normal hematopoietic stem cells and ASCs

Single cell culture was performed according to a previous study [17]. Briefly, individual cells isolated from different sources were placed into each well of 96-well microplates, ranging from 192 to 960 wells, as per the number of cells obtained from each patient (Additional file 2). Individual CD34 cells were cultured in serum-free medium containing 100 ng/mL stem cell factor, 100 ng/mL Flt-3, 100 ng/mL thrombopoietin, and 50 ng/mL granulocyte colony-stimulating factor (G-CSF) (all from Stem Cell Technologies, Vancouver, British Columbia, Canada). After culture for 5 days, each well of the microtiter plate was examined with an inverted microscope (Olympus IX50, Melville, NY) to determine growth and plating efficiency of the single CD34 cells. The growth and proliferative capacities of normal hematopoietic stem cells and ASCs were determined as a function of plating efficiency (the number of the wells in which more than two cells grew/total number of cells in 96-well plate culture × 100). Growth was quantified and graded with the following scoring system according to cell number in each CD34 clone: grade 1, 5 or less cells/well; grade 2, 6 to 10 cells/well; grade 3, 11 to 20 cells/well; grade 4, 21 or more cells/well.

### PCR array and real time PCR for the genes contributing to ASC quiescence

To screen for genes contributing to ASC quiescence, RNA (1 μg) extracted from ASCs (CD34+CD38- cells) and non ASCs (other CD34+ leukemic cell) isolated from BM samples obtained from a representative AML patient was converted to cDNA and amplified using the RT^2^ First Strand cDNA Synthesis Kit (SABiosciences, Frederick, MD, USA). The quality of cDNA was confirmed with the Human Stem Cell Signaling RT^2^ Profiler Array (SABiosciences), which tests for RNA integrity, inhibitors of reverse transcription and PCR amplification, and genomic and general DNA contamination [18]. Gene expression was then analyzed in these samples using the Human Stem Cell Signaling RT^2^ Profiler PCR Array (SABiosciences, PAHS-047), which profiles the expression of 84 genes involved in pluripotent cell maintenance and differentiation. PCR products were quantified by measuring SYBR Green fluorescent dye incorporation with ROX dye reference. Functional gene groupings consisted of the Hedgehog, Notch, TGF-b, and Wnt signaling pathways. PCR amplification was conducted on an ABI Prism 7500 sequence detection system, and gene expression was calculated using the comparative ∆∆Ct-based fold-change calculations from the uploaded raw threshold cycle data. Subsequenctly, aberrantly expressed genes were further confirmed by real time-PCR, using ASC and non-ASCs isolated from BM samples obtained from 7 AML patients.

### Analysis of mtDNA copy number in ASCs and designated AML cell populations

The mtDNA copy numbers were analyzed for the collected bulk cells from the CD34+CD38- cells (ASCs), CD34+CD38+ cells, CD33+ cells, and CD19+ cells. Total DNA was then extracted with an AccuPrep Genomic DNA Extraction Kit (Bioneer, Daejon, Korea). The extracted DNA was resuspended in TE buffer (10 mM Tris-HCl, 1 mM EDTA, pH 8.0) and photometrically quantified. The lysate was briefly microcentrifuged and stored at -20°C. A highly conserved region of the mtDNA genome that codes for the CYTB gene [nucleotide 14909 to nucleotide 15396; 488 base pairs (bp)] was selected to quantify the number of mtDNA copies. The PCR product of the CYTB gene was then subcloned into the pCR®2.1-TOPO® vector, and transformed into competent *E. coli* (TOP10 cells) using a TOPO TA cloning kit (Invitrogen). Quantitative PCR was conducted with a Rotor-Gene real-time centrifugal DNA amplification system (Corbett Research), at a final reaction volume of 25 μL containing 12.5 μL of 2 × QuantiTect SYBR Green PCR Master Mix (Qiagen), 0.4 μM each of the forward and reverse primers for the CYTB gene, 5 μL of template DNA (20 ng/reaction) or standard and RNase-free water. The mtDNA copy number of this calibrator was determined by dividing the total DNA concentration by the weight of each plasmid molecule. The length of the pCR®2.1-TOPO® vector was 3931 bp; thus, the cloned vector was a total of 4419 bp in length. After spectrophotometric determination of the plasmid DNA concentration (X), the copy number (Y) of the standard CYTB gene molecules was calculated using the following formula: X μg/μL plasmid DNA/4419 (plasmid length) × 660 × 6.022 × 10^23^ = Y molecules/μL. The molecular concentration of the plasmid stock solutions was diluted from 5.8 × 10^8^ copies to 5.8 × 10^5^ copies/μL, in order to generate the calibration curves. Thermal cycling conditions were as follows: one cycle of 50°C for 2 minutes and 95°C for 15 minutes, followed by 35 cycles of 94°C for 20 seconds, 56°C for 30 seconds, and 72°C for 30 seconds.

### The clinicopathological implications of the ASCs in AML patients

The clinicopathological parameters were evaluated as follows: age, sex, FAB classification, hemoglobin (Hgb), white blood cell count (WBC), platelet count (PLT), BM blast%, cytogenetic groups, the number of expired cases, the number of relapse cases, overall survival (OS) months, and relapse free survival (RFS) months. These parameters were analyzed by two groups according to the ASCs ratio (ASCs per total CD34+ cells) groups; group of low ASC ratio less than 0.1 (n = 27) and group of high ASCs ratio more than 0.1 (n = 33).

### Statistical analysis

Values of the average plating efficiency (%) of ASCs and variable normal HSCs were compared using the Mann-Whitney test for 2 groups and the Kruskal-Wallis test with Dunn’s multiple comparison correction was used for comparisons between 3 or more groups. The chi-square test was used to determine statistical differences in the parameters of two groups according to the ASCs ratio (ASCs per total CD34+ cells). OS time was defined as the time between diagnosis and death from any cause. RFS was defined as the time between diagnosis and relapse or disease progression from underlying disease. RFS and OS were estimated by the Kaplan-Meier estimate.

## Results

### Remarkable lower plating efficiency of ASCs than normal HSCs by single cell culture system

**Figure 1:**
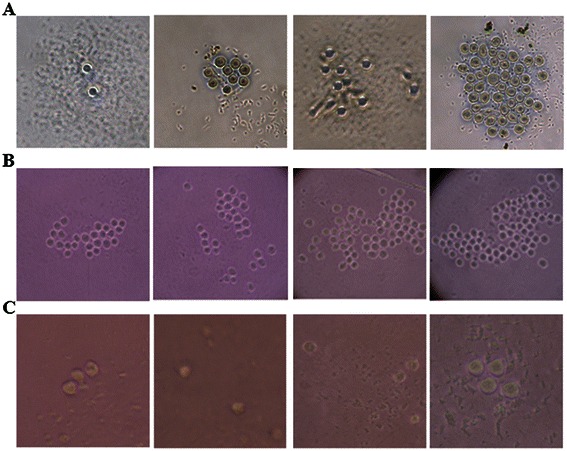
**Morphology of single hematopoietic and AML stem cell clones.** Single cells, either hematopoietic stem cells (HSCs) or ASCs, were placed in separate wells within 96-well plates and cultured in serum-free medium containing stem cell factor, Flt-3, thrombopoietin, and granulocyte colony-stimulating factor. After 5 days of culture, each well was examined using an inverted microscope to determine growth and plating efficiency of the single stem cell. **(A)** Normal HSCs obtained from adult bone marrow showed variable degree of proliferative potentials from Grade 1 to 4. **(B)** Almost all of HSCs obtained from cord blood showed high proliferative properties at Grade 4. **(C)** The plating efficiency of single ASCs remained at a remarkable low level at Grade 1. Detailed methods and analysis of plating efficiency of each single cell are described in the Methods section.

**Figure 2:**
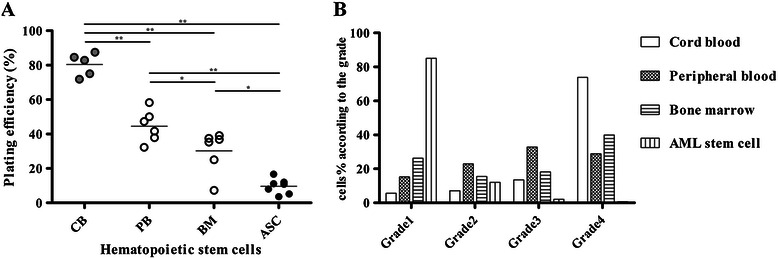
**Comparison of plating efficiency and proliferation capacities in single normal HSCs and ASCs.** The growth and proliferation capacities of single HSCs and ASCs were quantified and graded with the following scoring system according to the number of cells in each well of 96 microplate: Grade 1, ≤5 cells/well; Grade 2, 6 to 10 cells/well; Grade 3, 11 to 20 cells/well; and Grade 4, ≥21 cells/well. **(A)** The plating efficiency of single ASC was significantly lower than that of normal HSCs obtained from cord blood (CB), peripheral blood (PB), and adult bone marrow (BM). The plating efficiency of single HSCs varied among source samples. Values for plating efficiency of ASCs isolated from BM obtained from 7 AML patients are indicated with black circles; while those of normal HSCs in BM and PB obtained from healthy controls (n = 6) are indicated with white circles; and those for CB obtained from healthy controls (n = 5) are indicated with gray circles. **(B)** Almost all single CB cells showed high proliferative capacity of Grade 4; BM and PB stem cells showed similar grades of single cell plating efficiency. However, almost all single ASCs showed significantly lower dividing property at Grade 1. Statistical significance is indicated as follows: *, *p*-value < 0.05 and **, *p*-value < 0.01.

Figure 1 represented the morphology of the single-cell-derived clones originated from HSCs and ASCs. The proliferative properties of individual normal single hematopoietic stem cells varied according to the source of samples (Figure 1 and Figure 2B). When we subclassified the grade of plating efficiency, normal HSCs obtained from adult BM showed variable degrees of proliferative potentials with grade 1 to grade 4, however, almost all of HSCs obtained from CB had high proliferative properties with grade 4. Notably, ASCs showed remarkably low proliferative potentials (Figure 1 and Figure 2B). When we compared the plating efficiency of single HSCs and ASCs, CB showed the highest average plating efficiency at 79.0% (range, 71.9% to 87.5%; median, 82.9%). PB showed the second-highest plating efficiency at 45.3% (range, 32.3% to 58.3%; median, 44.5%) and BM stem cells followed third, at 31.1% (range, 7.3% to 39.1%; median, 36.1%). Of note, single ASC from AML patients showed a significantly lower plating efficiency, 8.6% (range, 3.6% to 16.7%; median, 10.9%) than did normal HSCs (CB, *p* = 0.0025; PB, *p* = 0.0012; and BM, *p* = 0.0221) (Figure 2A). These results directly confirmed the quiescent and slowly dividing properties of ASCs. In addition, the plating efficiency of normal HSCs varied by origin in the healthy donors.

### Identification of genes contributing to ASC quiescence

**Figure 3:**
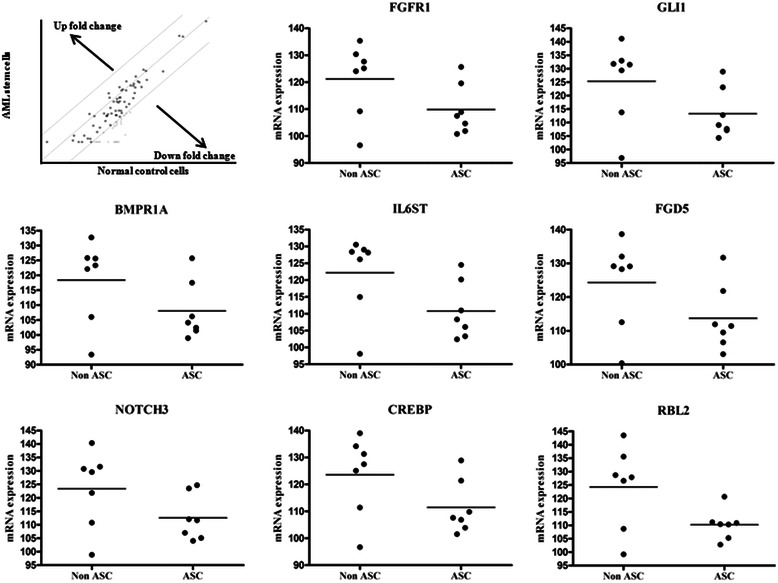
**Confirmation of genes showing significant down-regulated expression in ASCs.** Scatter plots revealed the expression status of genes showing down-regulation in ASCs and non-ASC leukemic cells. Initial screening for alterations in gene expression was performed using human stem cell signaling PCR array of samples from a representative AML patient and the results were further confirmed by quantitative real time PCR analysis of individual candidate genes by using samples obtained from 7 AML patients.

Of the 84 genes examined by human stem cell signaling profiler array, we found that the expression of 27 genes (32%) were persistently significantly decreased by >4-folds compared with that observed in non-ASCs (Additional file 3). Members of the TGFβ super-family signaling pathway (ACVR1C, ACVR2B, BMPR1A, BMPR2, CREBBP, E2F5, LTBP1, LTBP4, RBL2, SMAD2, SMAD3, SMAD9 and TGFBR1) were most commonly significantly decreased; as well as members of the Wnt (FZD3, FZD5, LRP6, NFATC4 and BCL9L); FGF (FGFR1, FGFR2, FGFR3); Notch (Notch 3, Notch 4 and RBPJL); pluripotency maintenance (IL6ST and LIFR); and hedgehog (GLI1) pathways. Among them, the expression of the eight genes i.e. fibroblast growth factor receptor 1 (FGFR1), GLI family zinc finger 1 (GLI1), bone morphogenetic protein receptor, type IA (BMPR1A), interleukin 6 signal transducer (IL6ST), frizzled family receptor 5 (FZD5), Notch 3, CREB binding protein (CREBP), and retinoblastoma-like 2(RBL2) had >10-fold decrease compared with that observed in counterpart non-ASCs (Table 1 and Figure 3).

**Table 1:** **Relative down-regulation of genes involved in proliferative activity in ASCs, as determined by PCR array and real-time PCR**

		Genes involved in proliferative activity							
		BMPR1A	CREBBP	RBL2	FZD5	FGFR1	Notch 3	IL6ST	GLI1
Fold-changes in gene expression (ASCs/non-ASCs)^*^		-12.8075	-11.6383	-11.3575	-12.5726	-14.0221	-12.309	-12.6345	-12.9973
Relative mRNA expression^†^									
	ASC 1	106.2	109.8	110.3	109.5	107.5	111.7	111.0	112.8
	ASC 2	101.4	106.9	111.2	111.9	104.7	107.0	106.1	109.1
	ASC 3	117.5	121.4	120.7	121.8	119.6	123.5	120.2	123.1
	ASC 4	98.9	103.9	102.8	106.6	101.9	104.0	103.3	107.2
	ASC 5	102.4	101.5	105.3	103.1	100.8	105.1	102.4	104.3
	ASC 6	125.7	128.9	110.9	131.7	125.7	124.7	124.5	128.9
	ASC 7	104.1	107.6	110.4	111.4	108.9	112.1	108.3	107.7
	Non ASC 1	125.8	125.1	128.7	128.3	125.2	129.6	128.4	131.5
	Non ASC 2	93.4	96.7	99.2	100.4	96.6	98.9	98.1	96.9
	Non ASC 3	125.6	134.2	127.9	132.0	130.4	131.6	128.2	133.0
	Non ASC 4	132.7	139.0	135.6	138.7	135.4	140.4	130.5	141.2
	Non ASC 5	106.0	111.4	108.7	112.6	109.2	110.8	115.0	113.8
	Non ASC 6	123.3	131.3	143.5	129.2	127.7	130.8	129.0	131.8
	Non ASC 7	122.1	127.5	126.6	129.1	124.1	121.9	126.2	129.4
	Average relative mRNA expression of ASCs	108.0	111.4	110.2	113.7	109.8	112.6	110.8	113.3
	Average relative mRNA expression of non ASCs	113.8	118.1	117.8	119.7	116.2	118.5	116.9	119.8

*Abbreviations*: *BMPR1A* Bone morphogenetic protein receptor, type IA, *CREBBP* CREB binding protein, *RBL2* Retinoblastoma-like 2, *FZD5* Frizzled family receptor 5, *FGFR1* Fibroblast growth factor receptor 1, *IL6ST* Interleukin 6 signal transducer, *GLI1* GLI family zinc finger 1.

^*^Fold-changes of down-regulated gene expression in ASCs relative to non-ASCs obtained from a representative AML patient.

^†^Relative mRNA expression was calculated as follows: 100 × threshold cycles of target/β-actin.

### Lower mtDNA copy number of ASCs than those of matched general leukemic cell populations

**Figure 4:**
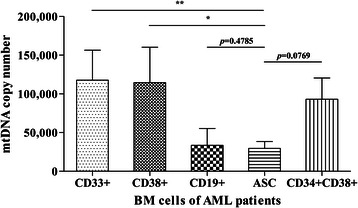
**The change of mtDNA copy number in each cell population from total bone marrow cells of AML.** Significant reduction of mtDNA copy number was observed in ASCs and CD19+ cell populations compared with other cells. There was statistical differences in mtDNA copy numbers from AML cells (CD33+ cells and CD38+ cells) and ASCs (CD34+CD38- cell) (*p* < 0.05), but the mtDNA copy number of ASCs was similar to those of normal control cells (CD19+). ASCs showed a lower mtDNA copy number than did non-ASCs (CD34+CD38+ cells), which was not statistically significant (*p* = 0.0769). Statistical significances were indicated as follows: *, *p*-value <0.05; and **, *p*-value <0.01.

There were no significant statistical differences between the ASCs (CD34+CD38-) and the CD19+ normal control cells (*p* = 0.4785). However, the mtDNA copy number of each sorted AML cell populations (CD33+ cells and CD38+ cells) were higher than ASCs (*p* = 0.0081 and *p* = 0.048). ASCs had a lower mtDNA copy number than non-ASCs (CD34+CD38+ cells), without statistically significant difference (*p* = 0.0769) (Figure 4).

### Clinical and laboratory implications of ASCs

**Figure 5:**
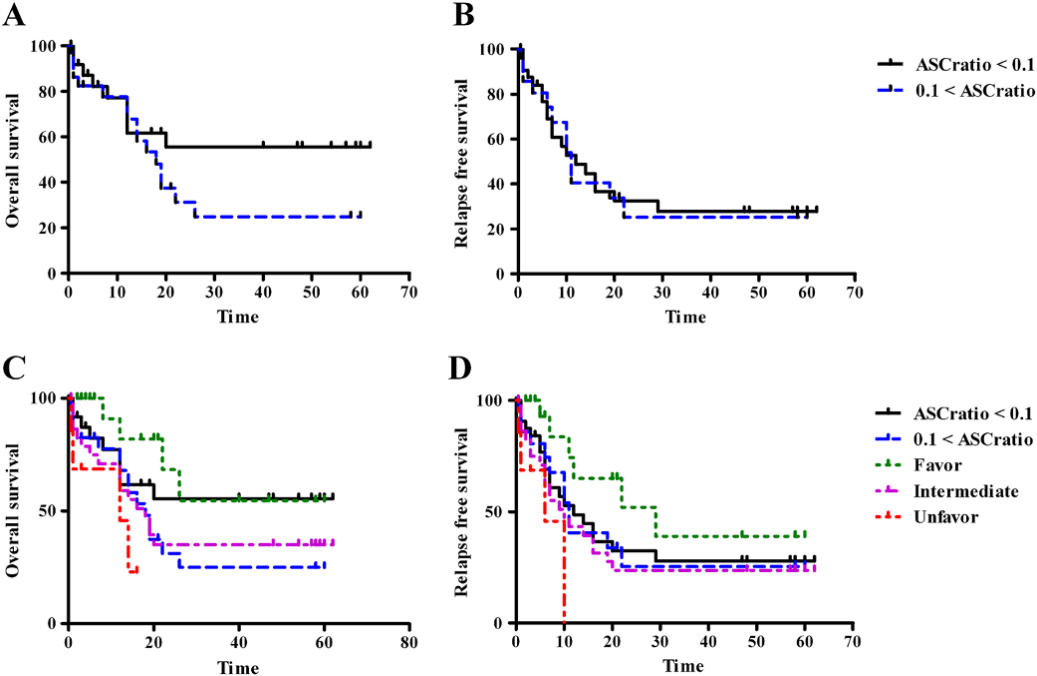
**Clinical implication of the proportion of ASCs. (A, C)** Compared to an ASC ratio of <0.1, an ASC ratio of >0.1 in the AML patients group resulted in a shorter overall survival, similar to that observed in cytogenetic risk groups, albeit without statistical significance (*p* = 0.211). **(B, D)** There was no statistically significant difference in relapse-free survival with respect to ASC ratio.

Patient demographics according to ASC ratio to total CD34+ cells were summarized in Table 2. There were no significant differences in sex, age, WBC, PLT, Hgb, BM blast%, and the number of expired or relapse cases. There were no significant differences also between groups according to FAB classifications, FLT3 mutation status, cytogenetic groups, and CD34%. However, the group of ASCs ratio more than 0.1 showed shorter OS than the group with ASCs ratio less than 0.1, but no statistical significance was noticed (median, 7 months vs 12 months; *p* = 0.211). When we analyzed OS according to the ASCs ratio and cytogenetic groups, the group of ASCs ratio with more than 0.1 showed similar prognosis with the unfavorable cytogenetic groups. The group of ASC ratio with less than 0.1, on the other hand, showed good prognosis similar to the favorable cytogenetic group (Figure 5). However, there were no statistically significant differences in RFS according to AML patient’s group with different ASCs ratio and proportion of total leukemic cells.

**Table 2:** **Demographics and clinical characteristics of AML patients according to ASCs ratio**

		Low ASC ratio (<0.1) group	High ASC ratio (>0.1) group	*p*-value
N		27	33	
Male/Female		14/13	14/19	0.466
Age, years	Min	15	16	0.471
	Max	81	82	
	Median (SD)	49 (3.2)	58 (3.2)	
FAB classifications, N (%)				0.724
	M0	2 (7.4)	1 (3.0)	
	M1	3 (11.1)	2 (6.1)	
	M2	15 (55.6)	19 (57.6)	
	M4	6 (22.2)	7 (21.2)	
	M5	1 (3.7)	2 (6.1)	
	M6	0 (0.0)	2 (6.1)	
Cytogenetics, N (%)^*^				0.621
	Favorable	9 (33.3)	9 (27.3)	
	Intermediate	16 (59.3)	19 (57.6)	
	Unfavorable	2 (7.4)	5 (15.2)	
CD34% groups, N (%)				0.031
	%CD34+ <5	2 (7.4)	7 (21.2)	
	5 ≤ %CD34+ <20	3 (11.1)	10 (30.3)	
	20 ≤ %CD34+	22 (81.5)	16 (48.5)	
WBC (*1000/uL)	Median	30.1	25.2	0.379
PLT (*1000/uL)	Median	37	44	0.698
Hemoglobin (g/dL)	Median	8.5	8.2	0.871
BM blast, %	Median	73	60	0.078
Expired case, N (%)		9 (33.3)	16 (48.5)	0.236
Relapse case, N (%)		14 (51.9)	17 (51.5)	0.979
Median overall survival, months		12	7	0.211
Median relapse free survival, months		7	6	0.442

ASCs ratio, the number of AML stem cell per the number of CD34+ cells; N, number; PLT, platelet; BM, bone marrow.

^*^Cytogenetic groups were divided according to the NCCN guideline [44].

## Discussion

This study presented evidence that ASCs obtained from the patients with AML showed significantly lower plating efficiency at the level of the single cell; this finding directly confirmed quiescent and slowly dividing properties of the ASCs by the single cell biological approach. We investigated the status of ASC mitochondria because, in addition to various cell-signaling pathways, this intracellular organelle is known to play an essential role as the main powerhouse in ATP generation and is implicated as the internal initiating center during apoptosis. Therefore, this study studied the change of mitochondrial genome in various cellular populations including CD34+CD38- cell population as well as CD34+CD38+ AML cells. ASCs had a significant reduction in mtDNA copy number, which may lead to decreased mitochondrial biogenesis and derangement of enzyme complex activities within the mitochondrial respiratory chain for ATP synthesis. These findings prompted us to investigate molecular alterations of ASCs compared with counterpart AML cells. The study using PCR arrays for genes involved in participating stem cell signaling pathways revealed remarkable down regulation of gene expressions in the important genes for maintaining stem cell stemness, self-renewal and proliferation. These molecular signatures which were revealed in single cell culture linked appropriately to unique properties of ASC cell biology and therapeutic targets of AML.

We assayed that individual normal single hematopoietic stem cells had variable proliferative capabilities and, above all, ASCs were the most dormant cells. This variation might be due to cellular environment-regulated stem cell quiescence, e.g. a bone marrow niche, as well as intrinsic molecular regulation of mandatory genes. The molecular crosstalk between HSCs and the cellular components of their niches was thought to control the balance between HSC self-renewal and differentiation [19]. Several recently identified genes that perturb HSC quiescence also disrupt stem cell maintenance and homeostatic blood cell production. It was suggested that the proliferative activity of HSC is normally restricted by both HSC intrinsic factors and extrinsic factors produced in the HSC niche [20]. ASCs had major clinical relevance due to their unique properties, such as slow mitosis, increased multidrug resistance and lower expression of Fas/Fas-L and Fas-induced apoptosis. ASCs are often resistant to both conventional chemotherapy and targeted therapies, are retained viable and contribute to relapse following discontinuation of therapy [21]. There has been increased interest recently to develop approaches based either on activating quiescent cancer stem cell to induce their cell cycle entry and increase their sensitivity to other treatments, or identifying agents that are capable of directly targeting quiescent cancer stem cells [21,22].

Although stem cells have the potential for self-renewal, they spend the majority of their time in the G0 phase of the cell cycle [23]. The quiescent feature of stem cells has been demonstrated in aspects of molecular signaling pathway, associated with cell cycle regulation. This study also found markedly declined expressions of the eight genes related to cell proliferation and differentiation. FGF signaling pathway was known to lead the loss of quiescence and depletion of the resident stem cell populations, which eventually diminishes regenerative capacity [24]. In Hedgehog signaling pathway, Merchant *et al*. revealed that the loss of the downstream effector Gli1 lead to reduced proliferation [25]. Notch is a crucial signaling pathway involved in the generation of cell diversity and stem-cell maintenance in different systems [26]. TGF-β signaling controls numerous cellular processes including cell proliferation, differentiation and apoptosis, both during embryogenesis and adulthood. The role of TGF-β in stem cell quiescence had been suggested not only in hematopoietic stem cells [27,28], but also in neural stem cells [29], and neonatal germ cell [30] with compelling supportive evidence. Evidence for a role of Wnt proteins in hematopoiesis arose from experiments demonstrating that multiple Wnts could expand hematopoietic stem/progenitor cells in culture [31]. A number of other genes and signaling pathways have been implicated in regulating stem cell quiescence as well [19].

Mitochondria play an essential role in ATP generation for cells and tissues, and is an internal center of apoptosis as well. Moreover, alteration of mitochondria and mtDNA sequence are now regarded as important causative factors for carcinogenesis, as well as metastasis. Therefore, we examined the mitochondrial genome in ASC and non-ASC populations. Primary AML cells, as non-ASC populations, had a significantly increased mtDNA copy number compared to ASC populations. In general, mitochondria has the major role in cell proliferation and differentiation with high requirement of ATP, causing increment of mtDNA copy number. It supposed that increased mtDNA copy number of non ASCs reflected active proliferation of leukemic cells. Excess mtDNA replications and increased mtDNA copy number are regarded as an initial event of pathological mitochondrial genome alteration; they may occur as a compensatory mechanism for mtDNA aberrations and mitochondrial dysfunction. Loss of mtDNA copy number in ASCs populations was likely due to either nuclear or mtDNA mutations [32]. Mitochondrial aberrations, including mtDNA somatic mutations and copy number variations, have been frequently reported in various human cancers [33-38]. However, the contents of mtDNA copy number could be influenced by various cancers in different manners. Lee *et al.* summarized that there is a significant reduction of mtDNA copy number in 57.4% (31/54) of the hepatocellular carcinoma, 54.8% (17/31) of the gastric cancers, 22.6% (7/31) of the lung cancers, and 28.0% (7/25) of the colorectal cancers compared with the corresponding non-tumorous tissues [39]. On the other hand, in breast cancer and colorectal cancer, increased mtDNA copy number was related to cancer risk [40,41]. Notably, variable mtDNA content had been reported as a prognostic factor for gastric cancer, colorectal cancer and non-small cell lung cancer [33,41,42]. These studies suggested that mtDNA copy number was closely related to not only tumorigenesis, but also regeneration of cancer cells as well.

Notably, the current study showed that the group with higher ASCs ratio (>0.1) had an unfavorable prognosis, albeit without statistical significance. This study, however, could not demonstrate any direct evidence of AML prognostic value with the ratio of ASCs. Several studies demonstrated that the quiescent, non-cycling state of ASCs may contribute to poor prognosis [4-6,9]. Conventional chemotherapeutic drugs that target leukemic cells have been shown to be ineffective in completely eradicating ASCs. The quiescent nature of ASCs might explain the low rates of long-term remission and multidrug resistance [43]. However, the prognostic value of ASCs may remain a matter of debate. Gerber *et al.* found that ASCs population could be divided according to the aldehyde dehydrogenase activity and CD34+CD38- fraction with high levels of aldehyde dehydrogenase activity was a potential marker for clinically significant minimal residual disease in AML [19]. Similar to our study, they also were not able to show the frequency of the ASCs as a surrogate prognostic marker in AML [19]. This might be caused by that ASCs population is heterogeneous, although CD34+CD38- cells are enriched for ASC. Further evaluation would be necessary to define this heterogeneity and clinical impact of ASCs.

## Conclusions

In conclusion, this study demonstrated the quiescence of ASCs with lower plating efficiency and slower dividing properties at the single cell level. This quiescence is represented as a marked decrease in the mtDNA copy number and also linked with down-regulation of genes in various molecular pathways. These findings might be used to improve the understanding of the molecular pathophysiology of AML as well as guide to novel treatment targeting ASCs.

## Additional files

Additional file 1: **Schematic flow chart of this study Single cell sorting and culture were performed for the evaluation of plating efficiency in the various sets of hematopoietic stem cells.** Briefly, individual CD34 cells placed into separate wells of 96-well plates were cultured in serum-free medium containing 100 ng/mL stem cell factor, 100 ng/mL Flt-3, 100 ng/mL thrombopoietin, and 50 ng/mL granulocyte colony-stimulating factor (G-CSF) (all from Stem Cell Technologies, Vancouver, British Columbia, Canada). After culture for 5 days, each well of the microtiter plate was examined with an inverted microscope (Olympus IX50, Melville, NY) to determine growth and plating efficiency of the single CD34 cells. Their molecular signatures related with quiescence were evaluated in terms of nuclear genomic changes and mtDNA copy number. The clinicopathological parameters in AML patients were also evaluated for the prognostic implication of ASC.
Additional file 2:
**Plating efficiency (%) of different hematopoietic stem cell sources and AML stem cells.**

Additional file 3:
**Human stem cell-signaling PCR array profiles for fold-down regulation genes in ASCs compared to non-ASCs obtained from a representative AML patient.**

## Abbreviations

AML: Acute myeloid leukemia
ASCs: AML stem cells
HSCs: Hematopoietic stem cells
mtDNA: Mitochondrial DNA
BM: Bone marrow
PB: Peripheral blood
CB: Cord blood
PBS: Phosphate-buffered saline
PE: Phycoerythrin
FITC: Fluorescein isothiocyanate
G-CSF: Granulocyte colony-stimulating factor
Hgb: Hemoglobin
WBC: White blood cell count
PLT: Platelet count
OS: Overall survival
RFS: Relapse free survival
