# Supplementary material for: Direct confirmation of quiescence of CD34+CD38- leukemia stem cell populations using single cell culture, their molecular signature and clinicopathological implications
Source: BMC Cancer. 2015 Apr 2;15:217. doi: 10.1186/s12885-015-1233-x (PMC4391681; doi:10.1186/s12885-015-1233-x)
Supplement: Additional file 1: — Schematic flow chart of this study Single cell sorting and culture were performed for the evaluation of plating efficiency in the various sets of hematopoietic stem cells. Briefly, individual CD34 cells placed into separate wells of 96-well plates were cultured in serum-free medium containing 100 ng/mL stem cell factor, 100 ng/mL Flt-3, 100 ng/mL thrombopoietin, and 50 ng/mL granulocyte colony-stimulating factor (G-CSF) (all from Stem Cell Technologies, Vancouver, British Columbia, Canada). After culture for 5 days, each well of the microtiter plate was examined with an inverted microscope (Olympus IX50, Melville, NY) to determine growth and plating efficiency of the single CD34 cells. Their molecular signatures related with quiescence were evaluated in terms of nuclear genomic changes and mtDNA copy number. The clinicopathological parameters in AML patients were also evaluated for the prognostic implication of ASC. [file 12885_2015_1233_MOESM1_ESM.doc]

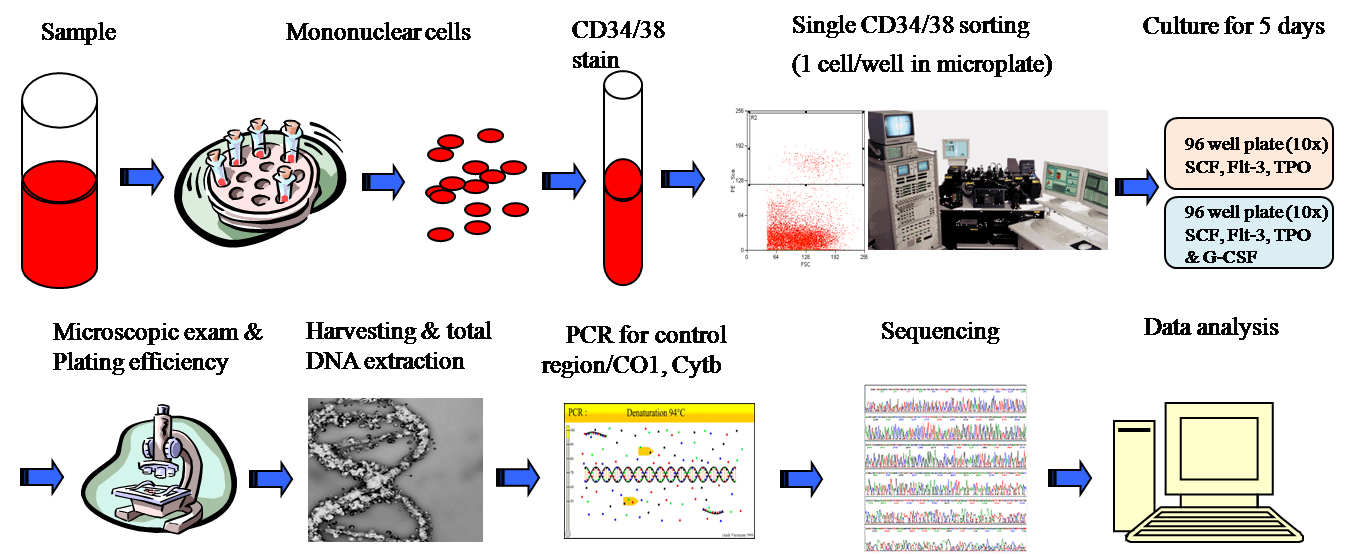


**Additional file 1 Schematic flow chart of this study Single cell sorting and culture were performed for the evaluation of plating efficiency in the various sets of hematopoietic stem cells.** Briefly, individual CD34 cells placed into separate wells of 96-well plates were cultured in serum-free medium containing 100 ng/mL stem cell factor, 100 ng/mL Flt-3, 100 ng/mL thrombopoietin, and 50 ng/mL granulocyte colony-stimulating factor (G-CSF) (all from Stem Cell Technologies, Vancouver, British Columbia, Canada). After culture for 5 days, each well of the microtiter plate was examined with an inverted microscope (Olympus IX50, Melville, NY) to determine growth and plating efficiency of the single CD34 cells. Their molecular signatures related with quiescence were evaluated in terms of nuclear genomic changes and mtDNA copy number. The clinicopathological parameters in AML patients were also evaluated for the prognostic implication of ASC.
