# Supplementary material for: Direct confirmation of quiescence of CD34+CD38- leukemia stem cell populations using single cell culture, their molecular signature and clinicopathological implications
Source: BMC Cancer. 2015 Apr 2;15:217. doi: 10.1186/s12885-015-1233-x (PMC4391681; doi:10.1186/s12885-015-1233-x)
Supplement: Additional file 2: — Plating efficiency (%) of different hematopoietic stem cell sources and AML stem cells. [file 12885_2015_1233_MOESM2_ESM.doc]

**Additional file 2. Plating efficiency (%) of different hematopoietic stem cell sources and AML stem cells**

| Specimens | Total no. of wells with single CD34+ cells | No. of wells showing more than 2 cells | Plating efficiency (%) |
| --- | --- | --- | --- |
| Bone marrow 1 | 192 | 75 | 39.1 |
| Bone marrow 2 | 480 | 35 | 7.3 |
| Bone marrow 3 | 960 | 339 | 35.3 |
| Bone marrow 4 | 960 | 240 | 25.0 |
| Bone marrow 5 | 960 | 360 | 37.5 |
| Bone marrow 6 | 960 | 354 | 36.9 |
| Peripheral blood 1 | 480 | 280 | 58.3 |
| Peripheral blood 2 | 384 | 192 | 50.0 |
| Peripheral blood 3 | 288 | 93 | 32.3 |
| Peripheral blood 4 | 480 | 200 | 41.7 |
| Peripheral blood 5 | 480 | 182 | 37.9 |
| Peripheral blood 6 | 480 | 227 | 47.3 |
| Cord blood 1 | 960 | 690 | 71.9 |
| Cord blood 2 | 480 | 406 | 84.6 |
| Cord blood 3 | 480 | 398 | 82.9 |
| Cord blood 4 | 480 | 420 | 87.5 |
| Cord blood 5 | 480 | 360 | 75.0 |
| AML stem cells 1 | 192 | 7 | 3.6 |
| AML stem cells 2 | 288 | 32 | 11.1 |
| AML stem cells 3 | 288 | 23 | 8.0 |
| AML stem cells 4 | 192 | 21 | 10.9 |
| AML stem cells 5 | 192 | 10 | 5.2 |
| AML stem cells 6 | 192 | 23 | 12.0 |
| AML stem cells 7 | 192 | 32 | 16.7 |
| Subtotal No. |  |  |  |
| Bone marrow | 4,512 | 1,403 | 31.1 |
| Peripheral blood | 2,592 | 1,174 | 45.3 |
| Cord blood | 2,880 | 2,274 | 79.0 |
| AML stem cells | 1,728 | 148 | 8.6 |
