# Supplementary material for: Direct confirmation of quiescence of CD34+CD38- leukemia stem cell populations using single cell culture, their molecular signature and clinicopathological implications
Source: BMC Cancer. 2015 Apr 2;15:217. doi: 10.1186/s12885-015-1233-x (PMC4391681; doi:10.1186/s12885-015-1233-x)
Supplement: Additional file 3: — Human stem cell-signaling PCR array profiles for fold-down regulation genes in ASCs compared to non-ASCs obtained from a representative AML patient. [file 12885_2015_1233_MOESM3_ESM.doc]

**Additional file 3. Human stem cell-signaling PCR array profiles for fold-down regulation genes in ASCs compared to non-ASCs obtained from a representative AML patient**

| Signaling pathway | Gene name (symbol) | Reference Sequences | Average Ct values | | Average ∆Ct | | Altered expression^*^ |
| --- | --- | --- | --- | --- | --- | --- | --- |
|  |  |  | non ASCs | ASCs | non ASCs | ASCs |  |
| TGFβ | Bone morphogenetic protein receptor, type IA (BMPR1A) | NM_004329 | 28.23 | 34.43 | 3.23 | 6.91 | -12.81 |
|  | CREB binding protein (CREBBP) | NM_004380 | 28.94 | 35.00 | 3.93 | 7.47 | -11.64 |
|  | Retinoblastoma-like 2 (p130) (RBL2) | NM_005611 | 28.97 | 35.00 | 3.97 | 7.47 | -11.36 |
|  | Latent transforming growth factor beta binding protein 4 (LTBP4) | NM_003573 | 29.40 | 35.00 | 4.40 | 7.47 | -8.43 |
|  | Activin A receptor, type IIB (ACVR2B) | NM_001106 | 28.51 | 33.54 | 3.51 | 6.02 | -5.70 |
|  | Activin A receptor, type IC (ACVR1C) | NM_145259 | 30.00 | 35.00 | 4.99 | 7.47 | -5.59 |
|  | Bone morphogenetic protein receptor, type II (serine/threonine kinase) (BMPR2) | NM_001204 | 30.15 | 35.00 | 5.15 | 7.47 | -5.01 |
|  | SMAD family member 2 (SMAD2) | NM_005901 | 28.79 | 33.56 | 3.79 | 6.04 | -4.75 |
|  | Latent transforming growth factor beta binding protein 1 (LTBP1) | NM_000627 | 27.89 | 32.63 | 2.88 | 5.10 | -4.65 |
|  | SMAD family member 9 (SMAD9) | NM_005905 | 30.34 | 35.00 | 5.33 | 7.47 | -4.41 |
|  | SMAD family member 3 (SMAD3) | NM_005902 | 28.27 | 32.89 | 3.27 | 5.37 | -4.28 |
|  | E2F transcription factor 5, p130-binding (E2F5) | NM_001951 | 28.83 | 33.42 | 3.82 | 5.89 | -4.20 |
|  | Transforming growth factor, beta receptor 1 (TGFBR1) | NM_004612 | 28.14 | 32.71 | 3.14 | 5.18 | -4.12 |
| Wnt | Frizzled family receptor 5 (FZD5) | NM_003468 | 28.83 | 35.00 | 3.82 | 7.47 | -12.57 |
|  | Low density lipoprotein receptor-related protein 6 (LRP6) | NM_002336 | 29.98 | 35.00 | 4.98 | 7.47 | -5.64 |
|  | Nuclear factor of activated T-cells, cytoplasmic, calcineurin-dependent 4 (NFATC4) | NM_004554 | 28.53 | 33.36 | 3.53 | 5.84 | -4.95 |
|  | Frizzled family receptor 3 (FZD3) | NM_017412 | 29.10 | 33.89 | 4.10 | 6.36 | -4.81 |
|  | B-cell CLL/lymphoma 9 (BCL9) | NM_004326 | 28.87 | 33.40 | 3.87 | 5.88 | -4.02 |
| Fibroblast Growth Factor (FGF) | Fibroblast growth factor receptor 1 (FGFR1) | NM_015850 | 28.67 | 35.00 | 3.66 | 7.47 | -14.02 |
|  | Fibroblast growth factor receptor 3 (FGFR3) | NM_000142 | 30.18 | 35.00 | 5.17 | 7.47 | -4.92 |
|  | Fibroblast growth factor receptor 2 (FGFR2) | NM_000141 | 28.98 | 33.73 | 3.98 | 6.21 | -4.69 |
| Notch | Notch 3 | NM_000435 | 28.86 | 35.00 | 3.85 | 7.47 | -12.31 |
|  | Notch 4 | NM_004557 | 28.49 | 33.17 | 3.49 | 5.64 | -4.47 |
|  | Recombination signal binding protein for immunoglobulin kappa J region-like (RBPJL) | NM_014276 | 29.72 | 34.24 | 4.71 | 6.72 | -4.00 |
| Pluripotency Maintenance | Interleukin 6 signal transducer (gp130, oncostatin M receptor) (IL6ST) | NM_002184 | 28.82 | 35.00 | 3.81 | 7.47 | -12.63 |
|  | Leukemia inhibitory factor receptor alpha (LIFR) | NM_002310 | 28.89 | 33.70 | 3.88 | 6.18 | -4.89 |
| Hedgehog | GLI family zinc finger 1 (GLI1) | NM_005269 | 28.78 | 35.00 | 3.77 | 7.47 | -13.00 |

*Altered expression of genes in ASCs was calculated using the comparative ∆∆Ct-based fold-change compared to non-ASCs.
